# Widespread use of proton pump inhibitors or potassium-competitive acid blocker has changed the status of gastrointestinal bleeding in patients with ischemic heart disease: real-world data from high volume centers

**DOI:** 10.1186/s12876-024-03269-w

**Published:** 2024-05-21

**Authors:** Shun Sasaki, Kazuhiro Ota, Makoto Sanomura, Yosuke Mori, Hironori Tanaka, Akitoshi Hakoda, Noriaki Sugawara, Taro Iwatsubo, Yuki Hirata, Kazuki Kakimoto, Hideaki Morita, Wataru Nagamatsu, Masaaki Hoshiga, Toshihisa Takeuchi, Kazuhide Higuchi, Hiroki Nishikawa

**Affiliations:** 1 Second Department of Internal Medicine, Osaka Medical and Pharmaceutical University, 2-7 Daigaku-machi, Takatsuki, Osaka 569-8686 Japan; 2 Department of Gastroenterology, Hokusetsu General Hospital, Takatsuki, Osaka Japan; 3 Department of Cardiology, Osaka Medical and Pharmaceutical University, Takatsuki, Osaka Japan; 4 Department of Cardiology, Hokusetsu General Hospital, Takatsuki, Osaka Japan

**Keywords:** Percutaneous coronary intervention, Gastrointestinal bleeding, Vonoprazan, Warfarin, Proton pump inhibitor

## Abstract

**Background:**

Although proton pump inhibitors (PPIs) or potassium-competitive acid blocker (PCAB) are useful in peptic ulcer prevention, their efficacy in preventing other gastrointestinal bleeding remains unclear. This study aimed to identify the status of gastrointestinal bleeding in the modern era when PPIs are widely used.

**Methods:**

This study included patients who underwent percutaneous coronary intervention (PCI) between 2018 and 2019 at two high-volume centers. Patients were categorized based on whether they experienced gastrointestinal bleeding within 2 years of PCI into groups A (patients who experienced gastrointestinal bleeding within 2 years after PCI) and B (patients who did not experience gastrointestinal bleeding).

**Results:**

Groups A and B included 21 (4.1%) and 494 (95.9%) patients, respectively (a total of 515 patients). Age at the initial PCI (77.8±2.4 and 72.0±0.5 years in groups A and B, respectively; *p* = 0.02), weight (53.8±3.2 and 61.8±0.7 kg in groups A and B, respectively; *p* = 0.01), and concomitant warfarin use (14.3% and 2.0% in groups A and B, respectively; *p* = 0.0005) were significantly different between the groups. The high bleeding risk rate (90.5% and 47.6% in groups A and B, respectively; *p* = 0.0001) was significantly different between the groups. A total of 95.9% of patients were taking PPIs or PCAB without significant differences between the groups. However, only one patient, who was taking steroids, had a gastric ulcer during PCAB treatment.

**Conclusions:**

Acid-related upper gastrointestinal bleeding is largely controlled by PPIs in post-PCI patients. Furthermore, the risk factors for non-acid-related bleeding include older age, lower weight, and concomitant warfarin use.

## Background

Patients with ischemic heart disease receive multiple antithrombotic agents post-percutaneous coronary intervention (PCI), and gastrointestinal bleeding is closely associated with mortality and ischemic complications in these patients [1]. Factors that cause gastrointestinal bleeding in post-PCI patients include a bleeding tendency and gastrointestinal mucosal injury due to antithrombotic medications and low-dose aspirin (LDA), respectively. However, not all patients experience gastrointestinal bleeding, and unknown factors, such as patient predisposition and the influence of concomitant medications, may exist. Recently, the risk of peptic ulcers has decreased, and the causes of gastrointestinal bleeding have changed due to the widespread use of proton pump inhibitors (PPIs), advent of vonoprazan [2], and decrease in *Helicobacter pylori* infection rates [3]. PPI use in post-PCI patients has recently increased compared with past practices, along with an increasing trend in vonoprazan use in this field. However, no current real-world data exists on how these trends affect gastrointestinal bleeding. Therefore, this retrospective study conducted at two high-volume cardiovascular medicine centers aimed to identify the risk factors for gastrointestinal bleeding development post-PCI and devise a strategy for its management. We believe that this real-world data may facilitate the development of preventive measures and lead to appropriate initial treatments for gastrointestinal bleeding in patients undergoing PCI in the future.

## Methods

### Patient population and study design

This retrospective study included patients who underwent initial PCI between January 2018 and December 2019 at two high-volume cardiovascular medicine centers in Osaka—the Osaka Medical and Pharmaceutical University Hospital and Hokusetsu General Hospital. Patients who could not be discharged from the hospital despite PCI due to the lack of improvement in their condition or death, those who had a history of malignant tumor (not involving intramucosal carcinoma after endoscopic treatment), and those who had inflammatory bowel disease were excluded from the study.

We used extractable items commonly found in the medical records of patients undergoing PCI for ischemic heart disease as parameters for consideration. The following parameters of each patient were evaluated before the initial PCI: age, sex, height, weight, comorbidities, medical information, drinking status, smoking status (Brinkman’s index), and blood test findings. The types and doses of antithrombotic medications and PPIs or potassium-competitive acid blocker (PCAB) used within 2 years post-PCI were also investigated. Based on the abovementioned factors, each patient was examined to determine if they met the criteria for high bleeding risk (HBR) rate (Japanese version) as defined by the Japanese Circulation Society (JCS) [4]. HBR was defined as having ≥ 1 or ≥ 2 of the primary and secondary factors, respectively. The primary factors included low body weight (< 55 and < 50 kg for males and females, respectively) or frailty, severe chronic kidney disease (estimated glomerular filtration rate (eGFR) < 30 mL/min/1.73 m^2^), anemia (hemoglobin (Hb) level < 11 g/dL), heart failure, long-term use of anticoagulants, peripheral arterial disease, history of non-traumatic bleeding, severe cerebrovascular disease, thrombocytopenia, active malignancy, cirrhosis with portal hypertension, chronic bleeding predisposition, major surgery that cannot be postponed during dual antiplatelet therapy (DAPT), and major surgery or trauma within 30 days before PCI. The secondary factors included older age (≥ 75 years old), moderate chronic kidney disease (eGFR 30-59mL/min/1.73m^2^), mild anemia (Hb levels of 11–12.9 and 11–11.9 g/dL for males and females, respectively), administration of non-steroidal anti-inflammatory drugs (NSAIDs) or steroids, and cerebrovascular disease.

“Gastrointestinal bleeding” was defined as any gastrointestinal bleeding requiring endoscopic hemostasis, hospitalization, or blood transfusion, or fatal gastrointestinal bleeding. When gastrointestinal bleeding occurred, the organs and diseases that were the sources of bleeding were investigated. Anemia was defined as < 11 g/dL, and any history of cerebrovascular disease, regardless of severity, was defined as fulfilling the major HBR criteria. We categorized the enrolled patients into the following groups to reveal the risk factors for gastrointestinal bleeding post-PCI: Groups A (patients who experienced gastrointestinal bleeding within 2 years post-PCI) and B (patients who did not experience gastrointestinal bleeding).

### Statistical analysis

Significant differences between groups were determined using the student’s t-test for continuous or categorical variables and Pearson’s chi-square test for binary variables. The predictors in the univariate analysis were analyzed using a multivariate logistic regression model to investigate the important risk factors for gastrointestinal bleeding. All reported *p*-values are two-sided. Statistical significance was set at *p* < 0.05. Data are expressed as mean±standard deviation. Odds ratios of continuous variables were analyzed using a logistic regression model. All statistical analyses were performed using JMP Pro 15.1 software (SAS Institute Inc., Cary, NC, USA).

### Ethics approval

This study was conducted in accordance with the 1975 Declaration of Helsinki (revised in 1983), and its protocol was approved by the Institutional Review Board of Osaka Medical and Pharmaceutical University Hospital (approval number: 2021-029). The study was publicized on the bulletin boards and websites of each facility to ensure that participants had the opportunity to refuse participation in the study.

## Results

Overall, 606 patients underwent the initial PCI at the participating hospitals within the survey period. Ninety-one patients met the exclusion criteria, among whom 24 could not be discharged from the hospital despite PCI because of lack of improvement in their condition or death; 65 had a history of malignant tumor of any state, including present malignant tumor; and 2 had inflammatory bowel disease. Ultimately, 515 patients were included in this study. Among these, 21 (4.1%) and 494 (95.9%) patients were in groups A and B, respectively.

### Risk factors for gastrointestinal bleeding after PCI

Table 1 shows the comparison between the characteristics of the patients in the two groups who underwent initial PCI. Significant differences were found in age (group A, 77.8±2.4 years; group B, 72.0±0.5 years; *p* = 0.02) and body weight (group A, 53.8±3.2 kg; group B, 61.8±0.7 kg; *p* = 0.01) but not in sex, height, Brinkman’s index, drinking status, dialysis, anemia calculated from Hb level, prothrombin time-international normalized ratio (PT-INR), concomitant NSAID use, or steroid use. No significant difference was found between the groups regarding whether the initial PCI was an emergency procedure for acute coronary syndrome. The prevalence of patients with HBR (Japanese version) was 90.5% and 47.6% in groups A and B, respectively, with a significant difference (*p* = 0.0001, odds ratio: 0.10).

**Table 1:** Characteristics of the patients in the two groups before the initial PCI

	Group A, *n* = 21	Group B, *n* = 494	Odds ratio (95% confidence interval)	*p*-value
Age at the initial PCI, years	77.8±2.4	72.0±0.5	1.06 (1.01–1.12)	0.02*
Male sex, n (ratio)	13 (61.9%)	346 (70.0%)	0.70 (0.28–1.71)	0.43
Height, cm	156.9±2.2	160.3±0.5	0.97 (0.93–1.01)	0.13
Weight, kg	53.8±3.2	61.8±0.7	0.96 (0.92–0.99)	0.01*
Brinkman’s index	304.8±147	489±30	0.999 (0.998–1.000)	0.18
Drinking history, n (ratio)	6 (28.6%)	151 (30.6%)	0.91 (0.35–2.39)	0.85
Dialysis, n (ratio)	1 (4.8%)	9 (1.8%)	2.69 (0.33–22.3)	0.34
Anemia, n (ratio)	4 (19.1%)	41 (8.3%)	2.6 (0.82–7.95)	0.20
PT-INR	1.22±0.11	1.09±0.02	1.44 (0.75–2.78)	0.27
Concomitant NSAID use, n (ratio)	0 (0%)	36 (7.3%)	-	0.20
Concomitant steroid use, n (ratio)	1 (4.8%)	17 (3.4%)	1.40 (0.18–11.1)	0.75
Initial PCI for ACS, n (ratio)	11 (52.4%)	217 (45.1%)	1.34 (0.56–3.21)	0.51
With HBR, n (ratio)	19 (90.5%)	235 (47.6%)	10.5 (2.41–45.4)	0.0001*

Patients who experienced any gastrointestinal bleeding within 2 years post-PCI were defined as group A, and others as group B. Data are expressed as mean±standard deviation unless stated otherwise. Anemia was defined as Hb < 11 g/dL. GI, gastrointestinal; PCI, percutaneous coronary intervention; ACS, acute coronary syndrome; HBR, high bleeding risk; PT-INR, prothrombin time-international normalized ratio; NSAID, non-steroidal anti-inflammatory drug; * indicates statistical significance

Table 2 shows the comparison between taking antithrombotic agents and PPIs or PCAB in the two groups after initial PCI. No significant difference was found in the duration of DAPT use (group A, 7.06±1.3 months; group B, 9.50±0.26 months; *p* = 0.07). A significant difference was found between the two groups in warfarin use (group A, 3/21 (14.3%); group B, 10/494 (2.0%); *p* = 0.0005, odds ratio 8.07), but not in anticoagulant use (group A, 6/21 [28.6%]; group B, 68/494 [13.8%]; *p* = 0.06) or any gastric acid secretion inhibitor use (PPIs or PCAB) (group A, 21/21 [100%]; group B, 473/494 [95.8%]; *p* = 0.33). Breakdown of the types of PPIs or PCAB used in group A was lansoprazole, vonoprazan, esomeprazole, and rabeprazole in 10/21 (47.6%), 8/21 (38.1%), 4/21 (19.1%), and 1/21 (4.8%), respectively, whereas, in group B, it was lansoprazole, vonoprazan, esomeprazole, and rabeprazole in 209/494 (42.3%), 145/494 (29.4%), 113/494 (22.9%), and 40/494 (8.1%), respectively. If a patient was taking several types of PPIs or PCAB due to a medication switch in the 2 years post-PCI, all medications were counted during this period. No significant difference was found between the two groups in terms of taking any dosage of vonoprazan, a PCAB (group A, 8/21 [38.1%]; group B, 145/494 [29.4%]; *p* = 0.39). A significant difference was found between the two groups in the use of 20 mg vonoprazan, which represents the maximum dosage of a gastric acid secretion inhibitor (group A, 4/21 [19.1%]; group B, 36/494 [7.29%]; *p* = 0.049, odds ratio: 2.99).

**Table 2:** Comparison of medications between the two groups after the initial PCI

	Group A, *n* = 21	Group B, *n* = 494	Odds ratio (95% confidence interval)	*p*-value
Duration of DAPT, month	7.06±1.3	9.50±0.26	0.02 (0.0004–1.2424)	0.07
Any anticoagulants, n (ratio)	6 (28.6%)	68 (13.8%)	2.51 (0.94–6.68)	0.06
DOACs, n (ratio)	3 (14.3%)	58 (11.7%)	1.25 (0.36–4.38)	0.31
Warfarin, n (ratio)	3 (14.3%)	10 (2.02%)	8.07 (2.04–31.9)	0.0005*
PCAB or any PPIs, n (ratio)	21 (100%)	473 (95.8%)	-	0.33
PCAB, n (ratio)	8 (38.1%)	145 (29.4%)	1.48 (0.60–3.65)	0.39
20 mg of vonoprazan, n (ratio)	4 (19.1%)	36 (7.29%)	2.99 (0.96–9.37)	0.049*

Patients who experienced any gastrointestinal bleeding within 2 years post-PCI were defined as group A, and others as group B. Data are expressed as the mean±standard deviation unless stated otherwise. GI, gastrointestinal; DAPT, dual antiplatelet therapy; DOAC, direct oral anticoagulant; PCAB, potassium-competitive acid blocker; PPI, proton pump inhibitor. * indicates statistical significance

Multivariate analysis was performed on the warfarin use and body weight based on the results of univariate analysis to identify patient characteristics and medication use that could serve as potential risk factors for gastrointestinal bleeding within 2 years after the initial PCI. The cut-off value of body weight was used based on the Japanese version of HBR as defined by JCS: low body weight was defined as < 55 and < 50 kg for males and females, respectively [4]. The multivariate analysis revealed that both warfarin use and low body weight (< 55 and < 50 kg for males and females, respectively) were independent risk factors for gastrointestinal bleeding within 2 years post-PCI (Table 3).

**Table 3:** Multivariate analysis to identify patient characteristics and medication use that can be potential risk factors for gastrointestinal bleeding within 2 years post-PCI

	Odds ratio (95% confidence interval)	*p*-value
Low body weight (< 55 and < 50 kg for males and females, respectively)	4.58 (1.76–11.9)	0.002*
Warfarin use	13.6 (3.09–60.3)	0.001*

PCI, percutaneous coronary intervention. * indicates statistical significance

### Breakdown of cases with gastrointestinal bleeding within 2 years post-PCI

Fewer cases of gastrointestinal bleeding were observed in this retrospective study than we had anticipated. Therefore, comprehensively examining each case was necessary, and Table 4 is presented. Twenty-one patients had gastrointestinal bleeding post-PCI. Four patients had colonic diverticular bleeding, three had ischemic colitis, two had rectal ulcer bleeding, and one patient each had reflux esophagitis, Mallory–Weiss syndrome, gastric ulcer, hemorrhagic gastric polyp, multiple small intestinal injuries, gastric angioectasia, small intestinal angioectasia, and colonic angioectasia. Two cases of bleeding after endoscopic resection of epithelial tumors—one each in the stomach and the cecum—were reported in patients who underwent warfarin therapy after aortic valve replacement. Although the bleeding disease in the remaining two cases could not be diagnosed, one was predicted to originate from the upper gastrointestinal tract and the other from the small intestine. Of the 21 gastrointestinal bleeding cases, not all required endoscopic hemostasis. Some cases were successfully treated with bowel rest through hospitalization and fasting or with medications, including mucosal defense factor enhancers. In cases of suspected small intestinal bleeding, capsule endoscopy was performed for diagnosis, and endoscopic hemostasis was not required. The results revealed a high rate of lower gastrointestinal bleeding, all patients were on PPIs or PCAB, and a high percentage of patients met the criteria for the Japanese version of HBR.

**Table 4:** Breakdown of the 21 cases of gastrointestinal bleeding within 2 years post-PCI

Disease	Age at initial PCI (years)	Sex	Height (cm)	Weight (kg)	Periods of DAPT use (months)	Concomitant anticoagulant therapy	PPIs or PCAB	Timing of bleeding (months post-PCI)	HBR	Hb (g/dL)	eGFR (mL/min/1.73 m^2^)	History of gastroduodenal ulcer	Others
Colonic diverticular bleeding	77	Male	160	60	3	-	VPZ 10 mg	3	age, anemia	11	62.6	-	
	75	Male	167.6	63.9	12	-	EPZ 20 mg	2	-	13.2	61.6	-	
	75	Female	151.2	46.1	0	-	VPZ 20 mg	7	age, CKD	7.6	27.0	Gastric ulcer	
	60	Male	177	80	6	-	LPZ 15 mg	1	-	13.2	60.6	-	
Ischemic colitis	76	Male	165.9	57.9	21	-	EPZ 10 mg	23	age, CKD	13.5	37.6	-	
	72	Female	145.3	49	6	-	LPZ 15 mg	3	weight	13.6	87.8	-	
	81	Male	159	44	12	-	LPZ 15 mg	11	weight, age, anemia	12.2	66.5	-	
Rectal ulcer	82	Female	133	35	2	-	LPZ 15 mg	23	weight, CKD, age	13.3	15.5	-	
	82	Male	161	48.3	0	-	EPZ 20 mg	0	weight, age, anemia	12	62.7	-	
Reflux esophagitis	70	Male	171.2	54.7	12	-	VPZ 10 mg	0	weight, anemia	10.6	79.7	-	
Mallory–Weiss syndrome	89	Female	144	36	3	-	LPZ 15 mg	4	weight, CHF, age, CVD	12.6	117.3	-	
Gastric Ulcer	81	Female	148	30.4	0	DOAC	VPZ 10 mg	6	weight, OAC, age, steroid	14.4	59.1	-	Taking 5 mg of prednisolone
Gastric polyp	68	Female	154.5	47.5	0	-	LPZ 15 mg	1	weight, CKD, anemia	10.1	6.6	-	
Small intestinal mucosal injury	78	Male	153.6	55.4	7	Warfarin	VPZ 20 mg	16	OAC, age, CKD, anemia	11	48.6	Ulcer after gastric ESD	PT-INR: 4.09
Gastric angioectasia	85	Female	143	42	6	-	VPZ 10 mg	1	weight, age, CKD, anemia	11.4	35.4	-	
Small intestinal angioectasia	74	Male	173.8	85	4	DOAC	LPZ 15 mg	4	OAC, CKD	14.3	54.0	-	
Colonic angioectasia	89	Female	141	36.5	0.5	DOAC	VPZ 20 mg	0.5	weight, OAC, age, CKD, anemia, CVD	11	34.9	Duodenal ulcer	
Bleeding after endoscopic treatment	79	Male	167.5	59.5	7	Warfarin	VPZ 20 mg	16	CKD, anemia, OAC, age	9.5	3.5	Ulcer after gastric ESD	Stomach PT-INR: 1.39
	77	Male	162.2	82.9	9	Warfarin	LPZ 15 mg	15	OAC, age, CKD, anemia	11.5	42.2	-	Cecum PT-INR: 1.94
Unknown bleeding origin	84	Male	160.7	47.8	6	-	EPZ 20 mg	8	weight, age, CKD, anemia	11	38.2	-	Suspected upper gastrointestinal bleeding
	80	Male	156	67	4	-	LPZ 15 mg	19	age, anemia	12.7	86.9	-	Suspected small intestinal bleeding

HBR, high bleeding risk defined by Japanese Circulation Society; DAPT, dual anti-platelet therapy; PCI, percutaneous coronary intervention; DOAC, direct oral anticoagulant; VPZ, vonoprazan; EPZ, esomeprazole; LPZ, lansoprazole; PT-INR, prothrombin time-international normalized ratio; CKD, chronic kidney disease; CHF, chronic heart failure; CVD, cerebral vascular disorder; ESD, endoscopic submucosal dissection; eGFR, estimated glomerular filtration rate

The results revealed a high rate of lower gastrointestinal bleeding, all patients were on PPIs or PCAB, and a high percentage of patients met the criteria for the Japanese version of HBR.

**Fig. 1:**
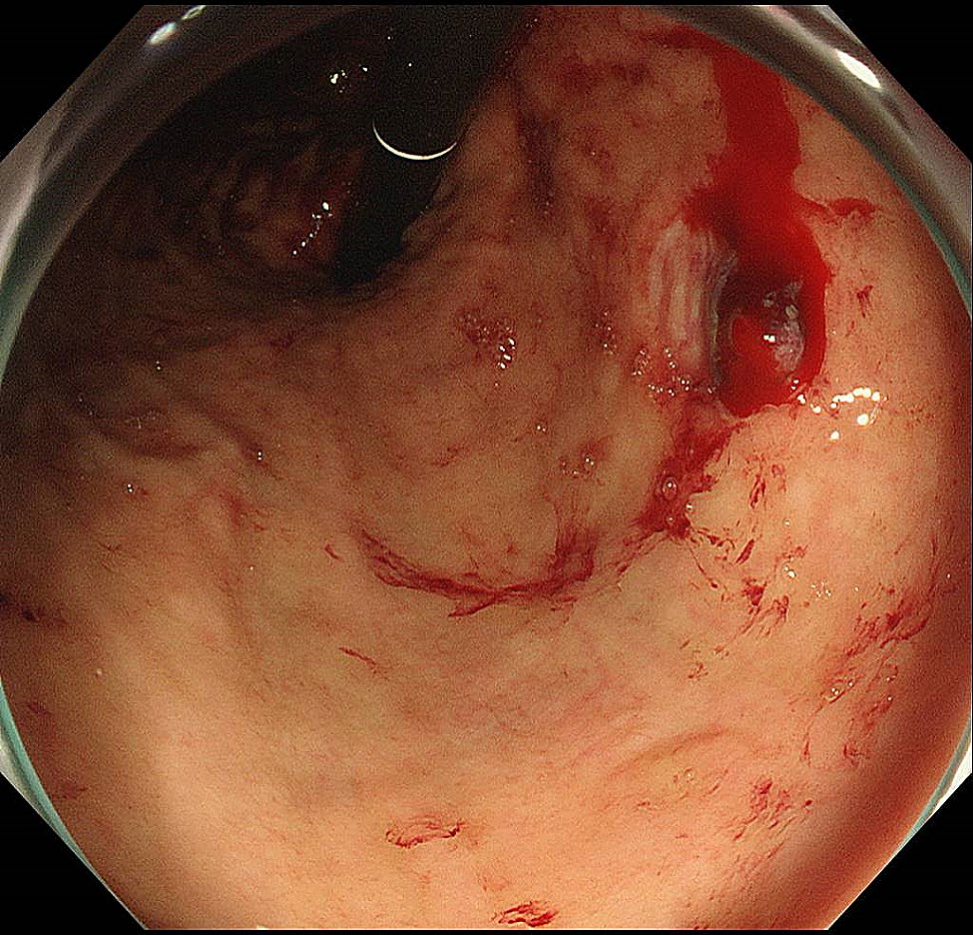
An 80-year-old female patient who was taking clopidogrel and a direct oral anticoagulant experienced a 10-mm ulcer in the lesser curvature of the gastric body with an exposed bleeding vessel despite concomitant use of 10 mg of vonoprazan. She was regularly taking 5 mg oral prednisolone for rheumatoid arthritis. After undergoing endoscopic hemostasis, the dose of vonoprazan was increased to 20 mg, and 300 mg/day of rebamipide, a gastric mucoprotective drug, was added to her medications. Three months later, the gastric ulcer was observed to be scarred

One case of hemorrhagic gastric ulcer occurred despite taking 10 mg of vonoprazan to prevent gastroduodenal mucosal injury. An 80-year-old female patient who was taking clopidogrel and a direct oral anticoagulant (DOAC) had a 10-mm ulcer in the lesser curvature of the gastric body with an exposed bleeding vessel (Fig. 1). She was regularly taking 5 mg of oral prednisolone for rheumatoid arthritis. After undergoing endoscopic hemostasis, the dose of vonoprazan was increased to 20 mg, and 300 mg/day of rebamipide, a gastric mucoprotective drug [5], was added to her medications. Three months later, the gastric ulcer was observed to be scarred.

A 70-year-old male patient presented with hematemesis due to severe reflux esophagitis on the day of emergency PCI of the left anterior descending branch. This condition appeared to have been caused by intense stress due to acute coronary syndrome. Reflux esophagitis occurred before the initiation of PPIs or PCAB and was cured after the patient started taking 10 mg of vonoprazan.

Among the patients who experienced gastrointestinal bleeding, three were taking warfarin medication: one with multiple small intestinal mucosal injuries and two following endoscopic treatment of epithelial tumors. In the case of small intestinal mucosal injury, the PT-INR at the time of bleeding was 4.09, which exceeded the optimal range. However, in the two post-endoscopic treatment cases, the PT-INRs were adequately controlled.

Four patients had a history of gastroduodenal ulcer; two had post-endoscopic gastric ulcer, and one of them had gastrointestinal bleeding post-PCI. One of the other patients had a gastric ulcer 3 years before the first PCI. The other patient had a duodenal ulcer scar in the duodenal bulb 12 years before the first PCI (Table 4).

## Discussion

To our knowledge, this is the first study to report the status of gastrointestinal bleeding in post-PCI patients since the revision of the Japanese guidelines on the diagnosis and treatment of acute coronary syndrome in 2018 and after the widespread use of vonoprazan and DOACs. The risk factors and characteristics of gastrointestinal bleeding were also examined to gather and assess real-world data.

PCI for ischemic heart disease is performed at many hospitals in Japan, and drug-eluting stents (DES) are most commonly used. DAPT is required for a certain period after DES implantation to prevent stent thrombosis. Concomitant anticoagulation therapy may be necessary for patients with arrhythmia, such as atrial fibrillation or flutter. Additionally, gastrointestinal bleeding may occur in patients taking antithrombotic medications. The acute coronary syndrome guidelines have been updated in 2018 and 2020, resulting in changes in the handling of antithrombotic medications [4, 6]. The 2020 JCS Guidelines Focused Update version, which is published by the JCS, presents the Japanese version of HBR assessment criteria. The primary criteria include low body weight, frailty, chronic kidney disease, and heart failure, among others, whereas the secondary criteria include patients aged ≥ 75 years and mild anemia, among others. The risk of bleeding increases if these criteria overlap. More than 50% of Japanese patients have HBR [4]. The flow chart for post-PCI antithrombotic therapy presented in this guideline was revised to begin with an assessment of bleeding risk. Additionally, the previously recommended duration of DAPT was reduced from 6 to 12 to 1–3 months in patients not receiving oral anticoagulant treatment or with low thrombotic risk. Furthermore, purinergic signaling receptor Y_12_ inhibitors, such as clopidogrel, are currently available as single antiplatelet therapy (SAPT) after DAPT [4].

Patients who meet the abovementioned criteria are recommended to receive PPIs for peptic ulcer prophylaxis. In a previous prospective study, the incidence of peptic ulcers in patients taking LDA was reportedly only 1.1% with esomeprazole—a PPI [7]. A systematic review and meta-analysis of previous studies analyzing the relationship between concomitant PPI use and gastrointestinal bleeding in post-PCI patients receiving clopidogrel-based DAPT reported that concomitant PPI use with DAPT was superior in preventing gastrointestinal bleeding, and half-dose PPI use was reportedly exhibited a lower risk of gastrointestinal bleeding than histamine H2-receptor inhibitor combination therapy. However, data are scarce, and which PPIs reduce the risk of gastrointestinal bleeding remains unclear [8]. Nakamura et al. reported an increased risk of gastrointestinal bleeding when anticoagulation agents were used for nonvalvular atrial fibrillation in Japanese patients receiving antiplatelet therapy post-PCI. Moreover, older age, lower body weight, and renal dysfunction are associated with bleeding risk [9]. Our previous report on LDA-induced small intestinal mucosal injury showed that approximately half of the post-PCI patients taking LDA with or without a PPI had a small intestinal mucosal injury, even in the absence of bleeding symptoms such as hematemesis [10].

A large joint prospective registry study between the United States and China has shown that starting PPIs earlier post-PCI prevents gastrointestinal bleeding during hospitalization. Although the study does not delve into the details of the bleeding organs, it argues that controlling upper gastrointestinal bleeding with PPIs reduces overall gastrointestinal bleeding [11]. A 10-year observational study in India on gastrointestinal bleeding incidence associated with LDA-based antithrombotic therapy with DAPT and SAPT revealed the risk factors for gastrointestinal bleeding and also reported that long-term antiplatelet use increased bleeding, mainly in the lower gastrointestinal tract [12]. Although the report examines gastrointestinal bleeding in patients receiving antithrombotic therapy for various diseases, ours is novel because the gastrointestinal bleeding was followed up for approximately 2 years post-PCI.

Vonoprazan significantly reduces the incidence of gastric acid-related diseases due to its potent suppression of acid secretion [13, 14]. In our study, the administration rate of PPI or PCAB for aspirin-induced gastroduodenal mucosal injury prevention was > 95%. Administering PPIs or PCAB in post-PCI patients requiring anti-platelet therapy is well recognized and has adequately controlled gastric acid-related disease development. Consequently, only one case—which required daily oral steroids for rheumatoid arthritis—of gastrointestinal bleeding caused by gastric acid-related diseases (gastric ulcer) occurred despite the patient taking 10 mg of vonoprazan. Here, a significant difference was found between the two groups regarding the administration of 20 mg vonoprazan, which is the maximum dose of gastric acid secretion inhibitor, in univariate analysis (Table 2). Excessive gastric acid suppression by 20 mg of vonoprazan might have undeniably altered the intestinal microflora and affected the bleeding from the small and large intestine, although no significant Hb or hematocrit level reduction was observed in patients receiving LDA with an additional 10 mg of vonoprazan in our recent study [15]. However, it should not be concluded that 20 mg of vonoprazan administration is a risk factor for gastrointestinal bleeding because vonoprazan tends to be administered to high-risk patients with upper gastrointestinal bleeding, as shown in a previous study [16]. Patients with HBR, particularly those with concomitant use of steroids, NSAIDs, or other complications such as collagen disease, require 20 mg of vonoprazan. Although upper gastrointestinal bleeding can be controlled using PPIs and PCAB in such patients, a risk of lower gastrointestinal bleeding still exists. Moreover, whether 20 mg of vonoprazan negatively affects lower gastrointestinal bleeding remains unclear. Regarding the interaction between PPIs and clopidogrel, omeprazole attenuates the drug effect of clopidogrel, whereas other PPIs and PCAB do not affect clopidogrel [17]. Nevertheless, no patients were taking omeprazole in this cohort.

This study revealed that warfarin use was an independent risk factor for gastrointestinal bleeding post-PCI. In contrast, DOAC use was not a risk factor for gastrointestinal bleeding. Warfarin has some limitations, including frequent PT-INR monitoring and relative dose adjustments to maintain it within the appropriate therapeutic range and food and drug interactions [18]. DOACs have fewer drug interactions, and monitoring coagulation status is not required [18]. A recent study reported that the incidence of lower gastrointestinal bleeding has increased, partly due to colonic diverticular bleeding and angioectasia in older adults, and the addition of oral anticoagulation therapy increases the risk of lower gastrointestinal bleeding [19]. Considering this study’s results, lower gastrointestinal bleeding might be more common than upper gastrointestinal bleeding in the modern era, where PPIs are widely used.

A recent Japanese study reported that the ratio of lower gastrointestinal bleeding, particularly diverticular bleeding, in older adults is increasing [20]. In our study, four cases of diverticular bleeding were observed, which was the most common cause of gastrointestinal bleeding. Interestingly, none of the patients with diverticular bleeding post-PCI were administered anticoagulants. Diverticular bleeding can occur with or without DOAC administration post-PCI. Furthermore, two of the four patients with diverticular bleeding did not have HBR. Angioectasia is also common in older adults, accounting for 1–6% of gastrointestinal bleeding [21]. Older adults with cardiac disease have dilated capillaries because of persistent chronic low blood reflux in the gastrointestinal mucosa [22]. Here, three cases of intestinal angioectasia with gastrointestinal bleeding and two of the three patients were administered DOACs. Our previous cross-sectional study revealed that the prevalence of small intestinal mucosal injury in post-PCI patients was approximately 50%, including asymptomatic cases [10]; however, this present study shows that only one case (< 0.2%) with actual clinical problems was observed. Therefore, some cases of small intestinal mucosal injury may have occurred rather than bleeding.

Regarding upper gastrointestinal bleeding, the diseases varied widely. Mallory–Weiss syndrome and gastric polyp bleeding were probably caused by antithrombotic medications, which exacerbated the bleeding tendency. Recent studies have shown that anticoagulant use is a risk factor for post-endoscopic submucosal dissection bleeding, whereas age is not a significant factor [23, 24]. One case of gastric ulcer occurred; however, insufficient acid suppression was not considered because 10 mg of vonoprazan was approximately two times as effective in inhibiting acid secretion as regular PPI [25]. These appear to be a combination of various adverse conditions of steroid-induced intractable mucosal injury. Although the number of cases in this study was relatively small to be definitive, patients taking steroids might also experience peptic ulcers even when taking 10 mg of vonoprazan, indicating that PPIs or PCAB alone may have poor efficacy in some patients.

Twenty-two of the patients who did not have gastrointestinal bleeding were not taking PPI/PCAB medication, and nine of them had HBR. Previous studies reported that the risk of upper gastrointestinal bleeding is reduced to 30% when clopidogrel is combined with a PPI [8]. Lué et al. revealed that PPIs should be administered to patients taking LDA to prevent upper gastrointestinal bleeding, although this may increase the risk of small intestinal bleeding (particularly LDA-induced small intestinal mucosal injury) [26]. Several effective medications for LDA-induced small intestinal mucosal injury are available [27, 28]. Fortunately, gastrointestinal bleeding did not occur in these 22 patients, and PPI/PCAB should have been taken. Although lower gastrointestinal bleeding was more common in this present case, we do not believe that PPIs or PCAB pose a risk for lower gastrointestinal bleeding. Previous reports have indicated that the risk factors for lower gastrointestinal bleeding include age, NSAID, LDA, clopidogrel, and warfarin, whereas the presence or type of PPI is not a risk factor. The rate of PPI use in patients with lower gastrointestinal bleeding was only 26.8% [29]. Therefore, we believe that the high incidence of lower gastrointestinal bleeding in our study was not caused by an increase in the number of lower gastrointestinal bleeding cases due to PPI/PCAB but by a relative increase in lower gastrointestinal bleeding as PPI/PCAB decreased the number of upper gastrointestinal bleeding cases.

Here, low body weight was also a risk factor for gastrointestinal bleeding, aligning with the observation reported by the JCS-HBR that low body weight was a risk factor for bleeding. However, the reasons cited by the JCS-HBR were related to fall trauma and subcutaneous bleeding associated with frailty, which were different from gastrointestinal bleeding. Generally, low body weight increases the dose per body weight of medications and may overstate the antithrombotic effect.

This study has some limitations. First, it was a two-center retrospective study, and the number of gastrointestinal bleeding cases was significantly smaller than expected. Therefore, they could not be comprehensively analyzed. Since the survey is medical record-based, it may not capture all gastrointestinal bleeding events. Although we found some statistically significant gastrointestinal bleeding-associated factors, they might be the result of type II errors due to the relatively small number of gastrointestinal bleeding cases. Consequently, multicenter or prospective studies are necessary to obtain more accurate data. Second, the bleeding group (group A) in this study included only clinically problematic cases, which may have underestimated the risk of gastrointestinal bleeding since we extracted only clinically significant gastrointestinal bleeding cases. This study focused only on gastrointestinal bleeding, which warrants significant attention. Third, many values in the collected data were missing. In this study, the missing values were removed from the analysis. Fourth, the *Helicobacter pylori* status of the participants was unknown. Although this was not a major limitation for analyses because only one gastric ulcer case was included in this study, *Helicobacter pylori* status should be investigated in an accompanying large-scale study in the future. Fifth, in this retrospective study, the choice of the types of PPIs or PCAB was based on the attending cardiologist’s preference. Additionally, our study’s results suggested that patients who were at high risk of experiencing gastrointestinal bleeding may have tended to select PCAB.

## Conclusion

Our study shows that PPIs or PCAB prevent gastric acid-associated upper gastrointestinal bleeding in post-PCI patients requiring antithrombotic medication. Older age, lower weight, and concomitant use of warfarin were risk factors for gastrointestinal bleeding without gastric acid involvement. Whether 20 mg of vonoprazan has a negative effect on lower gastrointestinal bleeding needs to be further investigated. Post-PCI gastrointestinal bleeding induced by gastric acid-related disease, particularly in the upper gastrointestinal tract, is decreasing because of the widespread use of PPIs and PCAB. Therefore, improved countermeasures for lower gastrointestinal bleeding may further improve the prognosis of patients post-PCI, such as the co-administration of mucoprotective medications.

## Data Availability

The datasets used and analyzed during this study will be available from the corresponding author upon reasonable request.

## Abbreviations

PCI: percutaneous coronary intervention
LDA: low-dose aspirin
PPI: proton pump inhibitor
PCAB: potassium-competitive acid blocker
HBR: high bleeding risk
JCS: Japanese Circulation Society
eGFR: estimated glomerular filtration rate
Hb: hemoglobin
DAPT: dual antiplatelet therapy
NSAID: non-steroidal anti-inflammatory drug
PT-INR: prothrombin time-international normalized ratio
DOAC: direct oral anticoagulant
DES: drug-eluting stent
SAPT: single antiplatelet therapy
